# Two-year cross-sectional studies reveal that single, young MSMs in Shenzhen, China are at high risk for HIV infection

**DOI:** 10.1186/s12985-019-1189-6

**Published:** 2019-06-22

**Authors:** Dijing Jia, Jin Zhao, Yongjian Liu, Xiaolin Wang, Lei Jia, Tao Gui, Lin Chen, Chenli Zheng, Jingwan Han, Tianyi Li, Jingyun Li, Hanping Li, Lin Li

**Affiliations:** 1 Department of AIDS Research, State Key Laboratory of Pathogen and Biosecurity, Beijing Institute of Microbiology and Epidemiology, Beijing, 100071 China; 2 Shenzhen Center for Disease Control and Prevention, Shenzhen, 518055 Guangdong China

**Keywords:** HIV-1, Cross-sectional study, Phylogenetic analysis, Drug-resistance

## Abstract

**Background:**

Shenzhen City is a rapidly growing area with a large number of floating populations, thus making it difficult to control HIV. Serial cross-sectional studies are helpful for the prediction of epidemiological tendency. In this study, two parallel cross-sectional studies were compared to explore changes in HIV epidemiology in Shenzhen, China.

**Methods:**

Two hundred and fifty newly reported HIV-positive cases were randomly selected in Shenzhen City in 2013 and 2015. Socio-demographical information was collected with informed consent. Full-length *gag* and partial *pol* genes were amplified using nested RT-PCR followed by sequencing and phylogenetic analysis. The genotypes of anti-HIV drug resistance were also analyzed. The characteristics of the HIV epidemics of 2013 and 2015 were compared to identify patterns.

**Results:**

The proportion of single, young MSMs dramatically increased in 2015 compared to 2013. Many subtypes, including CRF07_BC (36.4%), CRF01_AE (34.1%), CRF55_01B (10.2%), B (6.4%), CRF08_BC (3.4%), CRF59_01B (0.9%), C (0.7%), D (0.2%), CRF68_01B (0.2%), CRF67_01B (0.2%), and unique recombinant forms (URFs, 7.3%), were identified. Close phylogenetic relationships between strains prevalent in Shenzhen and other areas of China was observed. No epidemic cluster confined to single, young MSMs was identified. 0.4 and 2.8% of the strains contained transmitted drug-resistant mutations in 2013 and 2015, respectively.

**Conclusion:**

Although the interval period is short, changes in HIV epidemiology in Shenzhen City are distinct. Frequent surveillance of HIV epidemics in Shenzhen City is thus necessary. Single, young MSMs have become a high-risk population for HIV infection and should be considered as focus population for HIV prevention and behavior intervention in Shenzhen City.

**Electronic supplementary material:**

The online version of this article (10.1186/s12985-019-1189-6) contains supplementary material, which is available to authorized users.

## Background

HIV epidemics have recently undergone a rapid shift in transmission profile in China. High-risk populations of HIV infection have changed several times from the initial injecting drug users (IDUs), former plasma donors, sexual workers, MSMs, to the general population [1]. Together with the shift in predominant transmission routes, major HIV subtypes in China have also changed from subtype Thai B, subtype C, CRF07_BC, and CRF08_BC to CRF01_AE [2]. Considering that HIV epidemics in China continue to evolve and thus it is important to improve its surveillance.

Shenzhen, which is located in Guangdong Province in southern China, is a rapidly growing area with a population of approximately 13.8 million. More than 80% of the populations of Shenzhen are ‘non-local residents’ who have household registrations in other regions. Hence, Shenzhen is a city with large-scale migrations of ‘floating’ individuals, which has become a major challenge in the prevention and control of HIV epidemics in China [3–6]. The migration of the floating populations always makes the intervention strategies for these groups ineffective [7, 8]. In recent years, the overall HIV prevalence among these migrants throughout China has been rapidly increasing [9, 10]. Another big problem caused by ‘floating’ populations is that they are often associated with local epidemics [9, 11] and changes in the distribution of circulating HIV-1 subtypes [12]. Therefore, it is necessary to characterize HIV epidemics in Shenzhen City.

Phylogenetic analysis, in combination with traditional epidemiological surveillance, is useful for describing the transmission dynamics among different populations [13–18]. Distinguishing high-risk populations and understanding the spreading dynamic of HIV strains among different populations would be meaningful for prevention. Cohort studies are a good method for serial surveillance; however, large populations are not feasible for cohort studies due to their related high cost and uncontrollable loss ratio at follow-up. Serial cross-sectional studies are helpful for the prediction of epidemiological trends. Furthermore, changes in HIV high-risk populations can be distinguished, which will be important for HIV prevention and behavior intervention. This study aimed to identify changes in the molecular epidemiology of HIV in Shenzhen City to facilitate the development of preventive strategies.

## Materials and methods

### Study subjects and specimens

This study was approved by the Ethical Review Board, Science and Technology Supervisory Committee of the Beijing Institute of Microbiology and Epidemiology. All of the newly reported HIV-positive and treatment-naïve cases identified in 2013 and 2015 in Shenzhen City were collected by the Shenzhen Center of Disease Control and Prevention. Two hundred and fifty cases were randomly sampled using the simple-random sampling method and enrolled into this study with informed consent. Epidemiologic background information was collected using specific questionnaires by trained interviewers. 10 mL of peripheral whole blood was obtained and the plasma was separated for extraction of viral RNA.

### HIV-1 RNA extraction, amplification, and sequencing

Viral RNA was extracted from 500 μL HIV-1 positive plasma specimens after being concentrated using a High Pure Viral RNA kit (Roche, USA). Viral full-length *gag* (nucleotides 790–2292 using HXB2 as calibrator) and partial *pol* genes encompassing the entire protease and partial reverse transcriptase regions (nucleotides 2085–5096 using HXB2 as calibrator) were amplified separately using reverse transcriptional nested PCR as previously described [19]. Briefly, the first round of PCR was fulfilled using a TaKaRa one-step RT-PCR kit, and the second round PCR using High Fidelity Taq (Invitrogen, USA) with primers and thermal cycling conditions as described elsewhere [19, 20]. Positive PCR products were subjected to Sanger sequencing after purification with a variety of internal specific primers (available on request).

### Editing, assembly, genotyping, and phylogenetic analysis of HIV-1 sequences

Potential contamination was excluded by comparing all sequence segments with all of the known sequences in the HIV database by BLAST search (http://hiv-web.lanl.gov/content/index). All of the sequenced fragments were amended, and *gag* and *pol* genes from the same patient were assembled based on the overlapping sequences. In the current study, bidirectional sequencing was performed, and the background noise was very clear/minimal. When a secondary peak of < 30% was observed, this peak was excluded. Once the secondary peak was > 30%, we considered this position as an ambiguous base. Finally, the consensus sequences from one patient were obtained. We were very cautious on the identification of URFs. No breakpoints within URFs were identified at the joint sites of the *gag* and *pol* genes. Finally, the consensus sequence per patient was obtained. HIV sequence quality was determined using an online Quality-Control software (https://www.hiv.lanl.gov/content/sequence/QC/index.html). HIV genotype was determined by submitting genes to the NCBI viral genotyping tool (http://www.ncbi.nih.gov/projects/genotyping/formpage.cgi). HIV genotypes were determined by combining the results of the two regions and further confirmed by phylogenetic analysis. For phylogenetic analysis, all our assembled *gag* and *pol* sequences were aligned to the reference sequences separately using MSCLE software.. The final alignment was checked visually and edited manually using the BioEdit software package (version 7.0.0; T. Hall, North Carolina State University, Raleigh, NC, USA). Evolutionary distances were computed using the Kimura 2 parameter method, including both transitions and transversions. Phylogenetic trees were generated using the neighbor-joining method with MEGA6.06 software package and maximum likelihood methods with PhyML3.0 software package. Detailed information on the references used in the current work is provided in Additional file 1: Table S1. The reliability of topologies was estimated by performing bootstrap analysis with 1000 replicates. Possible intertype recombination events were further proven using the online Recombination Identification Program (RIP; version 3.0; http://hiv-web.lanl.gov) and confirmed by jpHMM online software (http://jphmm.gobics.de/). The gene sequences were deposited to GenBank as accession numbers MH635658 - MH636002, MH632305 - MH632713.

### Drug resistance analysis

All of the assembled partial *pol* genes were submitted to the Stanford HIV Drug Resistance Database website. TDR mutations were identified using the WHO 2009 list of mutations for surveillance of TDR as implemented in the Calibrated Population Resistance tool (v5.0 beta) (http://hivdb.stanford.edu) [21].

### Statistical analysis

All of the data were double entered into Microsoft Excel 2007 (Microsoft; Redmond, WA, USA). Categorical variables were compared using GraphPad Prism 6 software package. For contingency tables containing more than two rows, rows with both items < 5% were merged before performing the chi-square test. For contingency tables containing two rows, Fisher’s exact test was performed.

## Results

### Socio-demographic characteristics of the participants

**Fig. 1:**
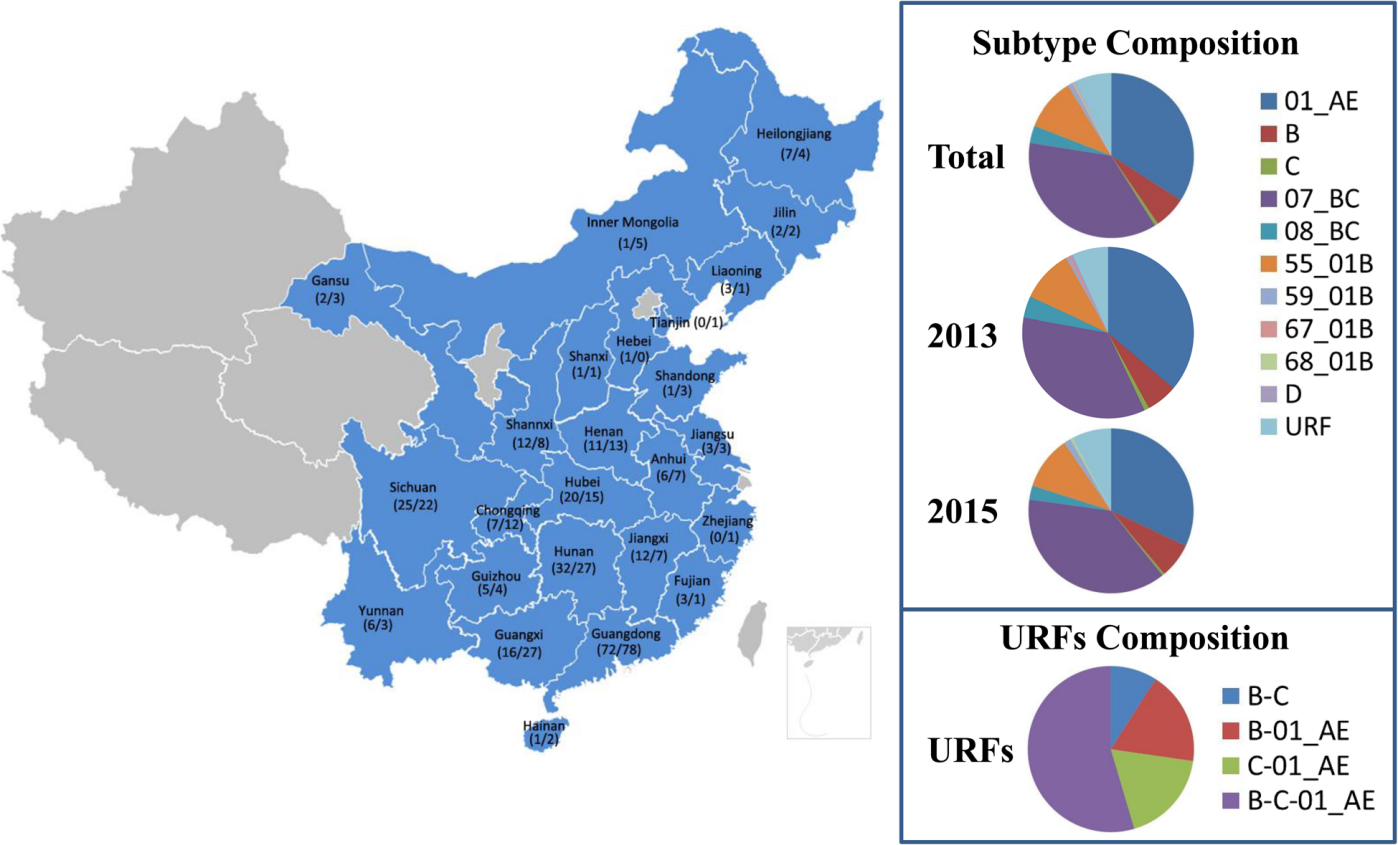
Geographical and subtype compositions of HIV positive cases identified in Shenzhen in 2013 and 2015. Map of China with prefecture names was listed in the left side. The numbers of HIV positive cases who registered the prefecture as hometown identified in Shenzhen in 2013 and 2015 are listed separately at the left and right of diagonal lines. Subtype composition of sequences was collected and depicted as a pie chart. Maps were generated using version 3.3.1 of the R package (https://www.r-project.org/)

Two hundred and fifty newly reported HIV-positive participants were enrolled in 2013 and 2015 separately. All of the subjects were not registered permanent residents of Shenzhen City. The geographic distribution of these HIV-positive ‘floating’ migrants are depicted in Fig. 1. The socio-demographic characteristics of 500 participants are summarized in Table 1. The median age of the participants was 35.1 years old in 2013 and 33.4 years old in 2015 separately (range: 19–57 years old in 2013 and 18–75 years old in 2015). Distinct shifts found in age distribution, transmission routes, and marital status were observed in the study cohort (*P* < 0.05). A higher number of young (< 30 years is defined as Age-Young and ≥ 30 years is defined as Age-Old) individuals were identified in 2015 compared to 2013 (*P* < 0.0001). In 2013, heterosexual transmission was the predominant route of HIV transmission. However, more MSMs than heterosexually transmitted HIV-positive individuals were identified in 2015 (*P* = 0.0174). In terms of marital status, the proportion of single individuals significantly increased in 2015 (*P* = 0.0017). Further detailed analysis showed that the proportion of single, young MSMs dramatically increased from 14.0% in 2013 to 26.4% in 2015. (*P* < 0.01) (Table 2).

**Table 1:** Social-demographic characteristics of participants

Characteristics	Case number (%)		
	2013	2015	*P*-value
Age (years)			0.001
2–14	0 (0.0%)	0 (0.0%)	
15–19	1 (0.4%)	8 (3.2%)	
20–25	36 (14.4%)	52 (20.8%)	
26–29	41 (16.4%)	63 (25.2%)	
30–34	65 (26.0%)	44 (17.6%)	
35–39	40 (16.0%)	22 (8.8%)	
40–49	48 (19.2%)	41 (16.4%)	
≥ 50	18 (7.2%)	20 (8.0%)	
Unknown	1 (0.4%)	0 (0.0%)	
Gender			> 0.05
Male	222 (88.8%)	221 (88.4%)	
Female	27 (10.8%)	29 (11.6%)	
Unknown	1 (0.4%)	0 (0.0%)	
Ethnic			> 0.05
Han ethnic	241 (96.4%)	234 (93.6%)	
Minor ethnic	9 (3.6%)	16 (6.4%)	
Marital status			0.002
Single	139 (55.6%)	161 (64.4%)	
Married	88 (35.2%)	53 (21.2%)	
Divorced, separated or widowed	23 (9.2%)	33 (13.2%)	
Unknown	0 (0%)	3 (1.2%)	
Education			> 0.05
College or higher	59 (23.6%)	76 (30.4%)	
Senior high school	77 (30.8%)	62 (24.8%)	
Junior high school	92 (36.8%)	93 (37.2%)	
Primary school or lower	22 (8.8%)	19 (7.6%)	
Occupation			> 0.05^a^
Catering services	6 (2.4%)	3 (1.2%)	
Official	7 (2.8%)	7 (2.8%)	
Worker	70 (28%)	65 (26%)	
Commercial service	61 (24.4%)	69 (27.6%)	
Unemployed	61 (24.4%)	78 (31.2%)	
Retired	3 (1.2%)	3 (1.2%)	
Migrant laborer	2 (0.8%)	4 (1.6%)	
Student	1 (1.6%)	2 (0.8%)	
Others	4 (0.4%)	12 (4.8%)	
Unknown	35 (14.0%)	7 (2.8%)	
Route of transmission			0.017
Heterosexual	133 (53.2%)	112 (44.8%)	
MSM	101 (40.4%)	130 (52.0%)	
IDU	12 (4.8%)	4 (1.6%)	
Blood-borne	1 (0.4%)	1 (0.4%)	
Unknown	3 (1.2%)	3 (1.2%)	

^a^Note: Unknown group was omitted before analysis

**Table 2:** Distribution of participants based on age, marital status and transmission route

Characteristics	Case number (%)	
	2013	2015
Age-Young (< 30 years old)	79 (31.6%)	123 (49.2%)
Transmission route (MSM)	36 (14.4%)	72 (28.8%)
Marital status (Single)	35 (14.0%)	66 (26.4%)
Marital status (Married)	1 (0.4%)	6 (2.4%)
Marital status (DSW^a^)	0 (0.0%)	0 (0.0%)
Marital status (Unknown)	0 (0.0%)	0 (0.0%)
Transmission route (Heterosexual)	42 (16.8%)	46 (18.4%)
Marital status (Single)	33 (13.2%)	39 (15.6%)
Marital status (Married)	9 (3.6%)	6 (2.4%)
Marital status (DSW^a^)	0 (0.0%)	1 (0.4%)
Marital status (Unknown)	0 (0.0%)	0 (0.0%)
Transmission route (Others)	1 (0.4%)	3 (1.2%)
Marital status (Single)	1 (0.4%)	3 (1.2%)
Marital status (Married)	0 (0.0%)	0 (0.0%)
Marital status (DSW^a^)	0 (0.0%)	0 (0.0%)
Marital status (Unknown)	0 (0.0%)	0 (0.0%)
Age-Old (≥30 years old)	171 (68.4%)	127 (50.8%)
Transmission route (MSM)	65 (26.0%)	58 (23.2%)
Marital status (Single)	42 (16.8%)	39 (15.6%)
Marital status (Married)	18 (7.2%)	12 (4.8%)
Marital status (DSW^a^)	5 (2.0%)	7 (2.8%)
Marital status (Unknown)	0 (0.0%)	0 (0.0%)
Transmission route (Heterosexual)	91 (36.4%)	66 (26.4%)
Marital status (Single)	22 (8.8%)	13 (5.2%)
Marital status (Married)	56 (22.4%)	27 (10.8%)
Marital status (DSW^a^)	13 (5.2%)	25 (10.0%)
Marital status(Unknown)	0 (0.0%)	1 (0.4%)
Transmission route (Others)	15 (6.0%)	3 (1.2%)
Marital status (Single)	8 (3.2%)	1 (0.4%)
Marital status (Married)	3 (1.2%)	1 (0.4%)
Marital status (DSW^a^)	4 (1.6%)	1 (0.4%)
Marital status (Unknown)	0 (0.0%)	0 (0.0%)

^a^*DSW* Divorced, separated, or widowed

### Distribution of HIV subtypes

A total of 345 full-length *gag* genes (69.0%) and 410 partial *pol* genes (82.0%) were successfully obtained from 500 samples. No evidence of sample contamination was found. Quality control of sequences showed that all of the gene structures were normal and with the correct open reading frames (ORFs). The subtypes of the HIV variants from 440 participants were finally determined. 160 strains were CRF07_BC (36.4%), 150 strains were CRF01_AE (34.1%), 45 strains were CRF55_01B (10.2%), 28 strains were subtype B (6.4%), 15 strains were CRF08_BC (3.4%), 4 strains were CRF59_01B (0.9%), 3 strains were subtype C (0.7%), 1 strain was subtype D (0.2%), 1 strain was CRF68_01B (0.2%), 1 strain was CRF67_01B (0.2%), and 32 strains were unique recombinant forms (URFs, 7.3%) (Fig. 1). The subtyping proportions in years 2013 and 2015 are summarized in Additional file 2: Table S2. The subtypes were further assessed using phylogenetic trees that were constructed using the sequences and the reference sequences representing subtypes A-D, F-H, J, K, and CRFs epidemic in China (including CRF01_AE, CRF07_BC, CRF08_BC, CRF31_BC, CRF55_01B, CRF57_BC, CRF59_01B, CRF61_BC, CRF62_BC, CRF64_BC, CRF65_cpx, CRF67_01B, and CRF68_01B), with subtype O as outgroup (http://www.hiv.lanl.gov, the reference information is summarized in Additional file 1: Table S1) (Fig. 4). By comparing the proportions of each subtype in 2013 and 2015, we found that the predominant strains shifted from CRF01_AE (36.10% vs. 31.96%) to CRF07_BC (34.84% vs. 37.90%). However, no statistical significance was observed (Fig. 1).

A total of 32 URFs were identified in Shenzhen City among newly reported HIV-positive individuals. The compositions of the genomes of each URF were further determined. Four patterns of genomes of URFs were distinguished, including B/C/CRF01_AE, C/CRF01_AE, B/CRF01_AE, and B/C recombinant forms. Considering that CRF01_AE and CRF07_BC are the predominant strains prevalent in Shenzhen City, it is unsurprising to find that the genomes of the highest proportion of URFs comprised B, C, and CRF01_AE (Fig. 1).

### Phylogenetic analysis of HIV sequences

To estimate the diversity of the sequences in this study within their respective subtypes, we calculated the genetic distances of different subtype sequences using the Kimura 2-parameter model based on the *gag* and *pol* genes (Table 3). The largest mean genetic distance was found in subtype B (8.6% ± 0.4%) within the *gag* gene. Relatively smaller mean genetic distances were found in CRF55_01B (3.9% ± 0.2%) and CRF07_BC (0.4% ± 0.2%).

**Table 3:** Genetic distances among sequences belonging to different subtypes

Gene	Genetic distance (mean ± SD)						
	CRF01_AE	CRF07_BC	CRF55_01B	B	CRF59_01B	CRF08_BC	C
*gag* (*n* = 313)	0.065 ± 0.003 (*n* = 128)	0.040 ± 0.002 (*n* = 113)	0.039 ± 0.002 (*n* = 37)	0.086 ± 0.004 (*n* = 21)	0.056 ± 0.006 (*n* = 2)	0.052 ± 0.004 (*n* = 10)	0.067 ± 0.007 (*n* = 2)
*pol* (*n* = 375)^a^	0.054 ± 0.003 (*n* = 131)	0.027 ± 0.002 (*n* = 152)	0.023 ± 0.002 (*n* = 45)	0.066 ± 0.004 (*n* = 26)	0.030 ± 0.004 (*n* = 4)	0.039 ± 0.003 (*n* = 14)	0.048 ± 0.005 (*n* = 3)

^a^ Only subtypes containing 3 or more sequences were included

**Fig. 2:**
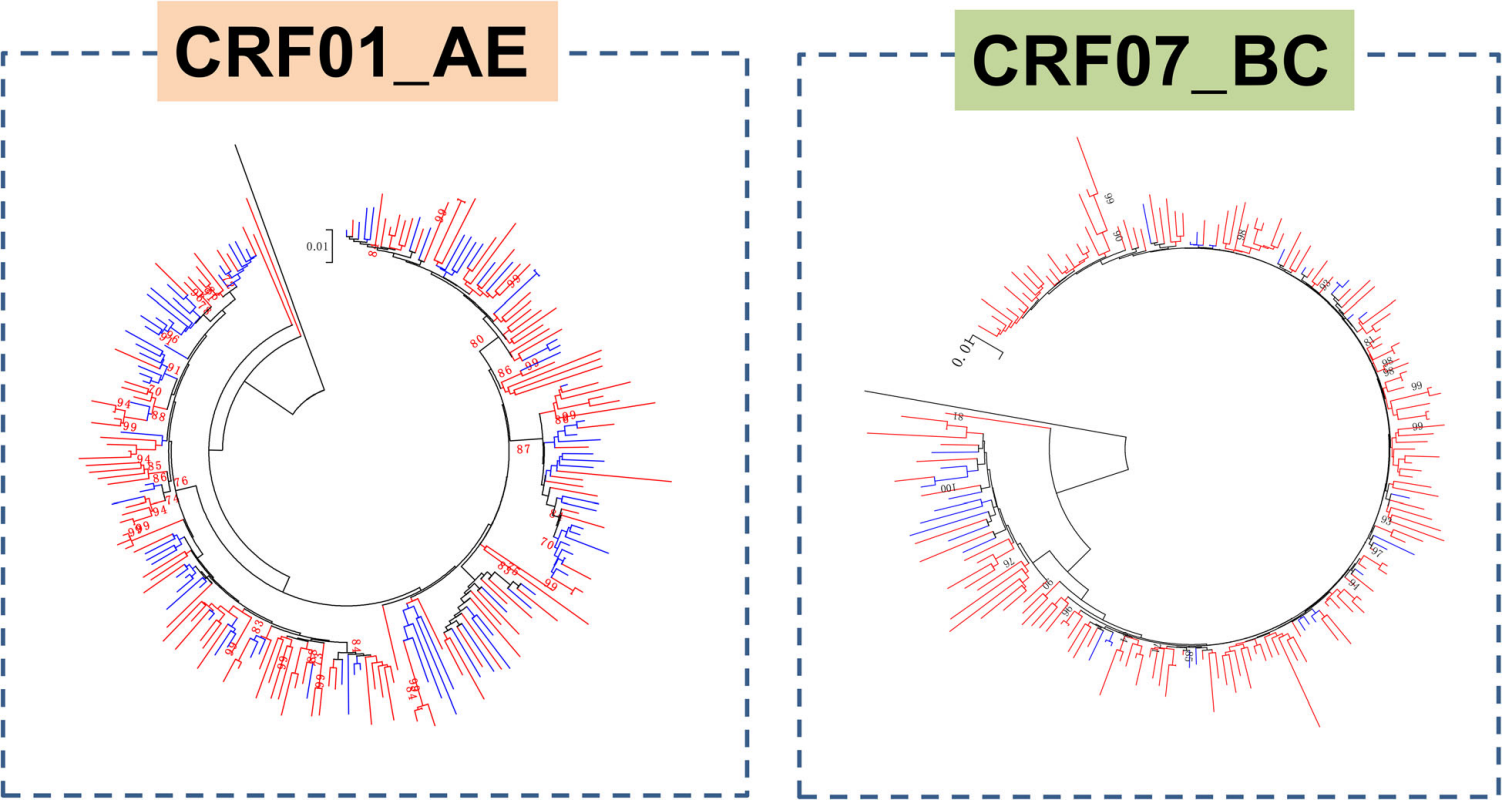
Maximum likelihood phylogenetic analysis of CRF01_AE and CRF07_BC *pol* sequences. Partial *pol* sequences representing CRF01_AE and CRF07_BC from different areas of China (blue color) were obtained from the database (http://hiv-web.lanl.gov/) and used for comparison with the sequences generated in this study (red color). Subtype J was used as outgroup

To explore the phylogenetic relationship of HIV strains with strains prevalent in other areas of China, we further aligned our sequences of CRF01_AE and CRF07_BC subtypes with references from the whole country. In the ML trees, the strains from Shenzhen were distributed in the phylogenetic trees equally, and no significant local cluster within the Shenzhen area could be identified (Fig. 2). The results revealed a close relationship between the HIV prevalence in Shenzhen and other areas. Therefore, the floating population in Shenzhen City might serve as a bridge for HIV epidemics that have spread to different areas in China.

**Fig. 3:**
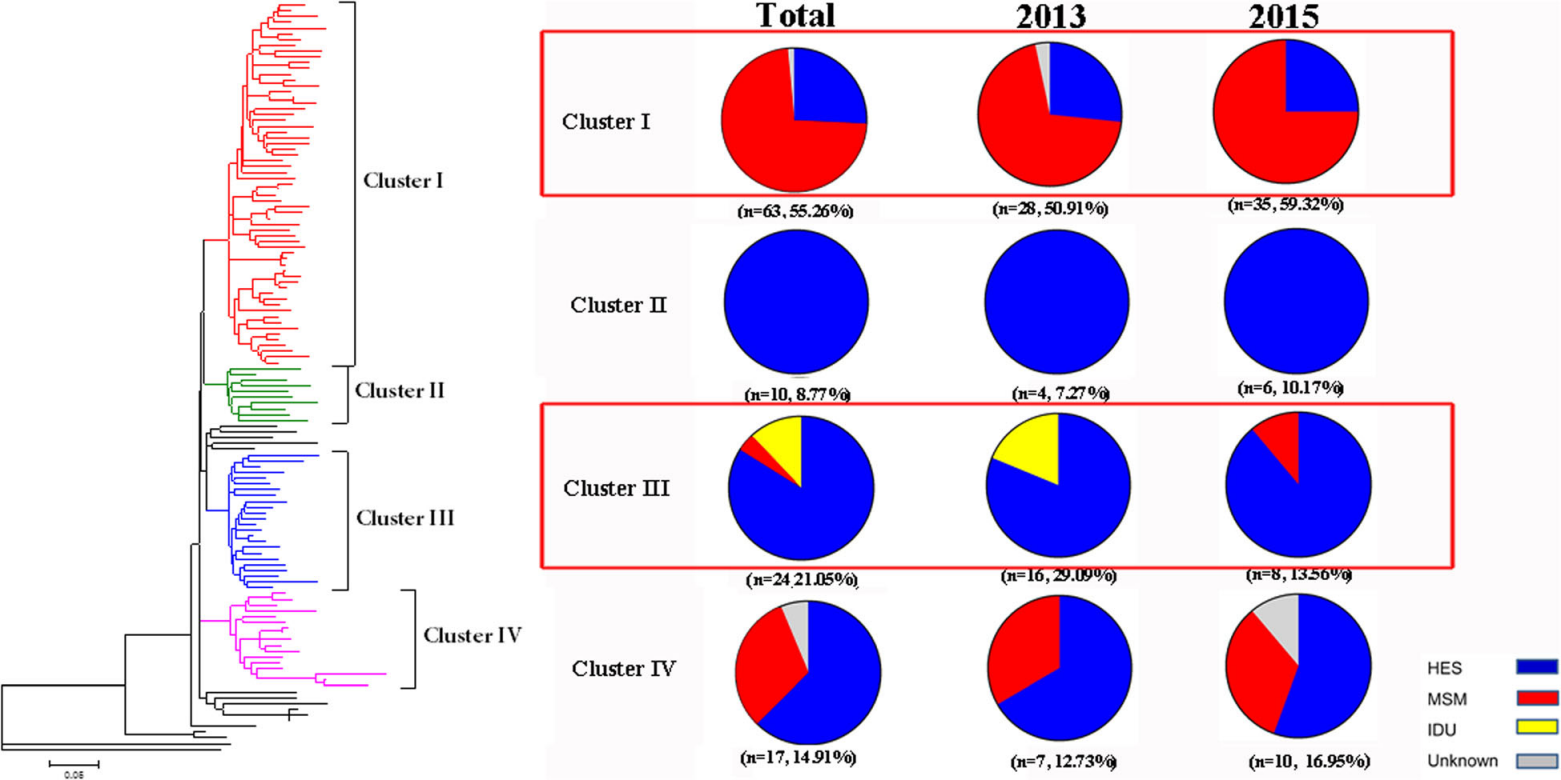
Compositions of four CRF01_AE clusters identified in Shenzhen City. Maximum Likelihood tree was created based on partial *pol* sequences of CRF01_AE identified in Shenzhen City. The background information of sequences in different clusters is summarized on the right side. Strains transmitted through different route are labeled in different colors as listed in the lower right corner

**Fig. 4:**
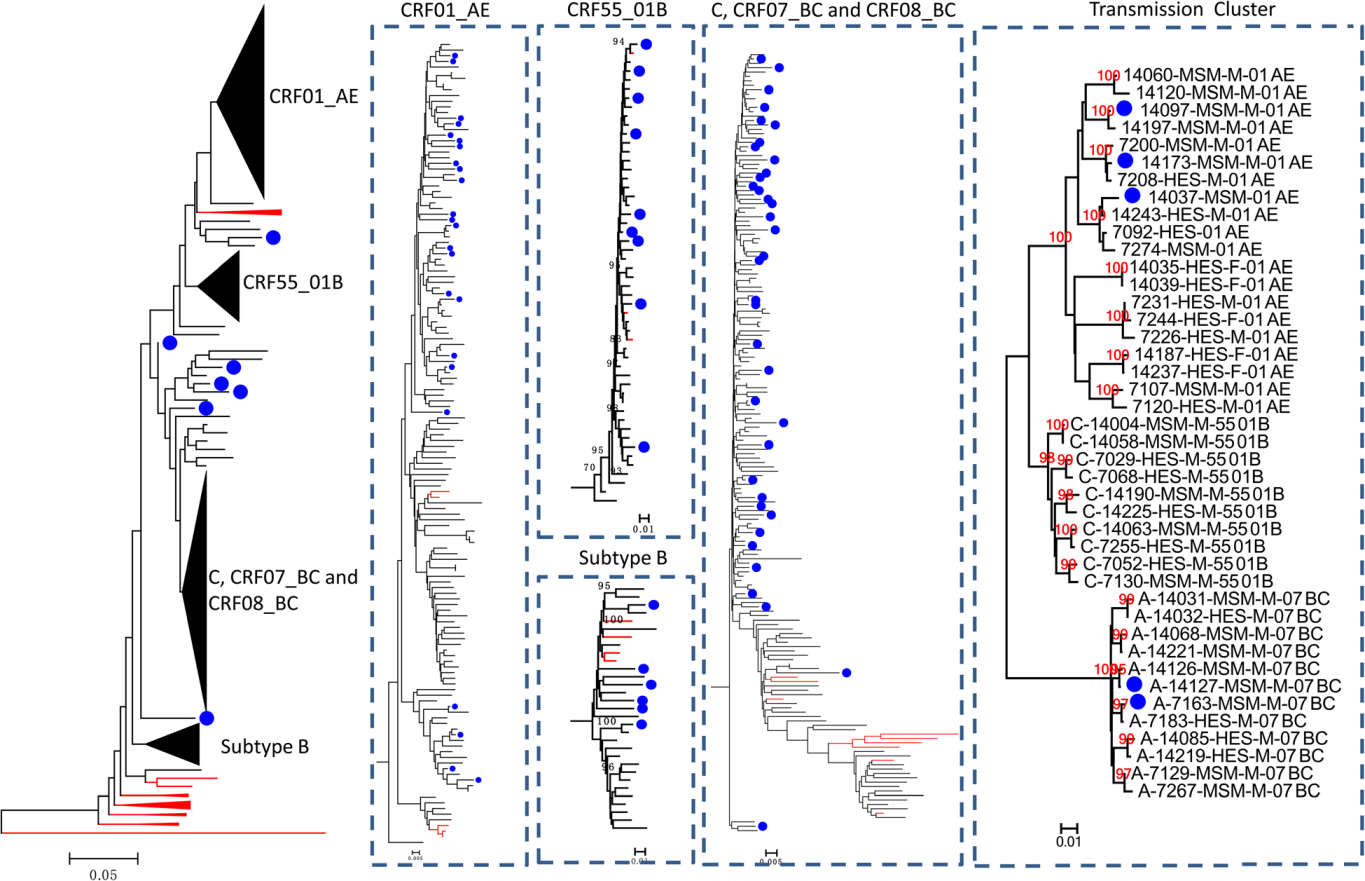
Phylogenetic analysis of HIV strains identified in Shenzhen city. Neighbor-joining tree was created with partial *pol* genes of our sequences and the reference sequences of subtypes A-D, F-H, J, K, CRF01_AE, CRF07_BC, CRF08_BC, CRF55_01B, CRF59_01B, CRF67_01B, CRF68_01B and group O (http://hiv-web.lanl. gov/). Each reference sequence is indicated by a red line. Sequences from single, young MSMs are labeled with a blue dot

Significant differences in topology structures of phylogenetic trees constructed with CRF01_AE and CRF07_BC sequences were observed. In the CRF07_BC tree, no large clusters containing more than 10 sequences were observed. However, in the CRF01_AE tree, at least four clusters were distinguished, with high bootstrap values. (Fig. 3). Cluster I (55.26%) is the most popular group, followed by clusters III (21.05%), IV (14.91%), and II (8.77%). Comparing the percentages of each cluster in 2013 and 2015 indicated that the percentage of cluster I increased from 50.91 to 59.32%, whereas cluster III decreased from 29.09 to 13.56%. Although no statistical significance was observed, the change in the ratio predict a change in CRF01_AE epidemic tendency in Shenzhen City. Further analysis of demographic information showed that the compositions of transmission routes of each cluster were significantly different (Fig. 3). Cluster I was mainly composed of sequences from MSMs (73.02%). Cluster II contained only strains obtained from heterosexual transmitted individuals (100%). Cluster III was mainly composed of strains from heterosexual transmission (83.33%) and IDUs (12.50%). Cluster IV was mainly composed of strains from heterosexual transmissions (58.82%) and MSMs (35.29%). Since the single, young MSMs became a high risk population for HIV infection in Shenzhen City, we further checked whether there were transmissions primarily confined to the population. The location of the sequences obtained from them was labeled in the phylogenetic tree (Fig. 4). No epidemic cluster that mainly comprised sequences from single young MSMs could be identified, indicating that there were no HIV strains that were confined to the specific population.

To understand the HIV transmission dynamics of young, single MSMs and other populations in Shenzhen City, we further evaluated the phylogenetic clustering and determined demographic, behavioral, and geographic characteristics of persons in transmission clusters that included young, single MSMs [22]. Clusters with bootstrap values higher than 95% and mean genetic distances less than 0.015 were selected as transmission clusters or pairs. A total of 20 transmission clusters or pairs were identified, which contained 44 sequences. Among all of the sequences, five sequences were from young, single MSMs. No transmission cluster or pair only containing young, single MSMs was identified. All of the young single MSMs were involved in clusters containing old MSMs or heterosexually transmitted individuals (Fig. 4). The viral diversity in the MSM population over time is presented in Additional file 2: Table S3.

### Drug resistance analysis

The 417 *pol* genes obtained in the study were submitted online to screen for transmitted drug resistance mutations as described in Materials and Methods. Drug resistance mutations were identified in two samples from 2013 (0.4) and seven samples from 2015 (2.8%), which were both below the WHO HIVDR Threshold Surveillance threshold of 5%, indicating that the local ART programs were functioning well. A total of nine mutations (three NRTI-resistant mutations, two NNRTI-resistant mutations, and four PI-resistant mutations) were identified and are listed in Table 4.

**Table 4:** Drug resistant mutations detected in newly reported HIV infected individuals in Shenzhen

ID	Gender	Transmission route	Viral Subtype	Drug-resistant mutations		
				PIs	NRTIs	NNRTIs
7137	M	HES	CRF01_AE	L76 V	–	–
7142	M	HES	CRF01_AE	–	L41I	–
14,003	F	HES	B	–	–	K101E
14,037	M	MSM	CRF01_AE	V82ILV	–	–
14,084	M	HES	CRF59_01B	F53 L	–	–
14,139	F	HES	CRF01_AE	–	–	L100I
14,146	M	HES	CRF07_BC	N83D	–	–
14,173	M	MSM	CRF01_AE	–	L74I	–
14,179	M	HES	CRF01_AE	–	V75 M	–

*PIs* protease inhibitors, *NRTIs* nucleoside reverse transcriptase inhibitors, *NNRTIs* non-nucleoside reverse transcriptase inhibitors

## Discussion

We compared the characterizations of newly reported HIV-positive cases in Shenzhen City between 2013 and 2015. A shift in HIV-positive populations was observed. Single, young MSMs were responsible for the observed higher number of HIV infections in 2015 compared to those in 2013. No HIV epidemic confined to single, young MSMs was identified. Close phylogenetic relationships among HIV strains between Shenzhen and other areas in China were observed both in 2013 and 2015. The results are important and helpful in terms of developing accurate preventive strategies for HIV control in Shenzhen City. It also highlights the importance of HIV surveillance, considering the rapid shifts in HIV-positive populations.

Public awareness, early initiation, and adherence to highly active antiretroviral treatment in index cases, and effective prevention strategies are all known to be important in the control of HIV epidemics. However, the vulnerable groups need to be specified as targets for intervention. It will be more cost-effective to use the limited HIV/AIDS resources available for target groups of people who are at the highest risk of infection. In this study, by comparing the characterizations of newly reported HIV-positive cases in Shenzhen City between 2013 and 2015, we have found that the population with the highest risk of HIV infection had changed. The proportion of single, young MSMs had significantly increased, underscoring the need to evaluate and intensify prevention efforts for this population.

Explore the origin of HIV epidemics in the target population is important for understanding the transmission dynamics, which in turn may provide clues for behavior intervention. Therefore, we further tested the relationship between HIV strains in epidemics in single, young MSMs and other populations. No epidemic or transmission clusters could be identified in single, young MSMs, indicating that there were no close connections among them. More interconnections might happen between single, young MSMs and old MSMs or heterosexually transmitted populations. Behavioral intervention confined only to single, young MSMs may be insufficient in controlling further transmissions. This undoubtedly will make it more difficult for HIV prevention in Shenzhen City. More comprehensive studies are necessary to distinguish individuals connected to the population.

Multiple subtypes of HIV were found in Shenzhen City. We identified 11 HIV subtypes or CRFs in Shenzhen City. The emergence of many subtypes of HIV in the same city might be due to its high level of floating population. The floating population brings back and forth the HIV strains prevalent in different areas of China. To some extent, the HIV epidemic in Shenzhen even can profile HIV epidemics in the whole country. In this study, we found that the ratio of CRF07_BC strains increased in 2015 compared to 2013. The tendency is in accordance with a previous study that focused on MSMs in Shenzhen City [23]. The results indicated that CRF07_BC might spread again in MSMs. In 2016, we conducted phylodynamic analysis of CRF07_BC strains prevalent in China, which showed that CRF07_BC had undergone a second rapid spread in China [24]. Therefore, further studies on the CRF07_BC epidemic in China are necessary. Multiple subtypes of HIV circulating in the same population always predict the emergence of recombinant. A high level of URFs (11.2%) was observed in Shenzhen City. In 2015, CRF55_01B was reported to be formed in Shenzhen City and rapidly spread in MSMs outside Shenzhen City [25]. Therefore, surveying HIV URFs formed in Shenzhen City will be helpful in the prediction of the emergence of CRFs in China.

In 2003, the government of China launched the “Four Free and One Care” policy [26]. Since then, HAART has been available. However, along with the extensive use of HAART, HIV-1 drug resistance has become an issue in some areas of China. In some individuals receiving therapy, the ratio of HIV drug-resistant mutations was higher than 20% in some areas [27]. In this study, a low level of drug-resistant strains was found in Shenzhen City both in 2013 and 2015. However, more drug-resistant variants were identified in 2015 compared to 2013, suggesting that the transmission of drug resistance has increased. Therefore, surveillance of HIV drug resistance is necessary in the region.

This study has some limitations. The two cross-sectional studies are based on newly reported HIV positive cases collected in 2013 and 2015. The time period is only 2 years, which is relatively short for monitoring changes in large HIV-positive populations. However, some shifted characterizations of HIV-positive populations have been selected. If the time is long enough, then more changing characteristics on HIV epidemiological trends could be distinguished and may explain why no statistical significance was observed on HIV subtype distribution and even transmitted drug resistant mutations. In addition, although all of the participants were newly reported HIV positive cases, it is difficult to estimate the exact infection time. Hence, it was considered that the mean interval time for an individual to be detected after being infected was the same between 2013 and 2015.

## Conclusions

In conclusion, we observed that the proportion of single, young MSMs significantly increased in newly reported HIV-positive cases in Shenzhen City, which will provide additional information for HIV prevention and behavior intervention. The identification of many HIV subtypes circulating in Shenzhen City will provide more information that may be utilized for HIV vaccine design. Furthermore, the close phylogenetic relationship of HIV strains between Shenzhen and many other areas in China suggests that the Shenzhen HIV epidemic might be useful for the prediction of HIV prevalence in the entire country. This study also highlights the importance of more comparable cross-sectional studies, which will be cost-effective for surveillance of the HIV epidemic in China.

## Additional files

Additional file 1: **Table S1.** Detailed information on the references used in this study. (XLSX 14 kb)

Additional file 2: **Table S2.** Subtyping proportions in 2013 and 2015. **Table S3.** Viral diversity in 2013 and 2015. (DOCX 15 kb)

## Data Availability

All of the data generated or analyzed during this study are included in this published article.

## Abbreviations

CRFs: Circulating recombinant forms
HIV: Human immunodeficiency virus
IDUs: Injecting drug users
jpHMM: jumping profile hidden Markov model
MSM: Men who have sex with men
URFs: Unique recombinant forms
