# Supplementary material for: Two-year cross-sectional studies reveal that single, young MSMs in Shenzhen, China are at high risk for HIV infection
Source: Virol J. 2019 Jun 22;16:83. doi: 10.1186/s12985-019-1189-6 (PMC6589171; doi:10.1186/s12985-019-1189-6)
Supplement: Supplementary file 2 — Table S2. Subtyping proportions in 2013 and 2015. Table S3. Viral diversity in 2013 and 2015. (DOCX 15 kb) [file 12985_2019_1189_MOESM2_ESM.docx]

**Table S2.** Subtyping proportions in 2013 and 2015.

| Subtype | Proportion | |
| --- | --- | --- |
|  | **2013** | **2015** |
| CRF01_AE | 36.20% | 31.96% |
| B | 5.88% | 6.85% |
| C | 0.90% | 0.46% |
| CRF07_BC | 34.84% | 37.90% |
| CRF08_BC | 4.07% | 2.74% |
| CRF55_01B | 9.95% | 10.50% |
| CRF59_01B | 0.45% | 1.37% |
| CRF67_01B | 0.45% | 0.00% |
| CRF68_01B | 0.00% | 0.46% |
| D | 0.45% | 0.00% |
| URF | 6.79% | 7.76% |
| Total | **100%** | **100%** |

**Table S3.** Viral diversity in 2013 and 2015.

| **Subtype** | **Gene Region** | **Diversity** | |
| --- | --- | --- | --- |
|  |  | **2013** | **2015** |
| CRF01_AE | *gag* | 0.055 | 0.052 |
|  | *pol* | 0.044 | 0.028 |
| CRF07_BC | *gag* | 0.032 | 0.031 |
|  | *pol* | 0.019 | 0.019 |
| CRF55_01B | *gag* | 0.031 | 0.027 |
|  | *pol* | 0.018 | 0.021 |
| B | *gag* | 0.097 | 0.093 |
|  | *pol* | 0.066 | 0.066 |
